# Monocyte‐Platelet Aggregates Are Major Source of BDNF after Bacterial Stimulation of Human Peripheral Blood Immune Cells

**DOI:** 10.1002/eji.202451538

**Published:** 2025-04-01

**Authors:** Fabien Sarcletti, Marco Dijmarescu, Michael Eigenschink, Nadja Wukowits, Barbara Oehler, Tanja Mayer, Sarah Pell, Anastasia Tandecki, David Seki, Andreas Spittler, David Berry, Angelika Berger, Lukas Wisgrill

**Affiliations:** ^1^ Division of Neonatology, Pediatric Intensive Care and Neuropediatrics Department of Pediatrics and Adolescent Medicine Comprehensive Center for Pediatrics Medical University of Vienna Vienna Austria; ^2^ Joint Microbiome Facility of the Medical University of Vienna and the University of Vienna Vienna Austria; ^3^ Department of Microbiology and Ecosystem Science Centre for Microbiology and Environmental Systems Science University of Vienna Vienna Austria; ^4^ Core Facility Flow Cytometry & Department of Surgery Research Lab Medical University of Vienna Austria

**Keywords:** bacterial stimulation, BDNF, monocyte‐platelet aggregates, short‐chain fatty acids

## Abstract

The gut microbiota and the immune system are closely connected, influencing early‐life brain development. Brain‐derived neurotrophic factor (BDNF), crucial for neuronal development, has been demonstrated to be produced by certain immune cells. However, the modulation of BDNF during bacterial antigen and metabolite challenge remains elusive. We investigate the effects of bacterial‐derived antigens and metabolites on BDNF secretion in human PBMCs. Although BDNF levels were altered during stimulation, a specific cellular origin of BDNF within PBMCs was indeterminate. Positive magnetic separation of monocytes eliminated both the stimulant‐induced BDNF secretion and reduced monocyte‐platelet aggregates. Conversely, elevated platelet counts significantly increased BDNF levels, indicating that platelets, when interacting with monocytes and exposed to bacterial antigens, are likely the dominant source of BDNF in PBMC cultures. As previously described, platelets are a crucial source of circulating peripheral blood BDNF. Our findings emphasize the importance of the interplay between immune‐blood cell complexes during microbial stimulation in regulating BDNF levels. This highlights the necessity of investigating such interactions to better understand the early‐life gut‐brain axis.

## Graphical abstract

Bacterial antigens primarily induce BDNF release from platelets interacting with monocytes in PBMCs. This interplay underscores how immune‐blood cell complexes shape BDNF levels which may impact early human development.

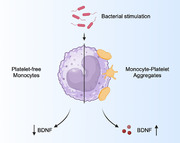

## 1 Introduction

Alterations in brain development and subsequent brain injuries, including intraventricular haemorrhage, periventricular leukomalacia and cerebellar haemorrhage, are major complications of extremely premature infants [1]. Preterm birth can lead to aberrations in brain development regarding the total brain volume, the cortical surface area and the microstructural organization [2]. Various growth factors and signalling molecules contribute to the healthy development of the human brain. One group of structurally related proteins in the central and peripheral nervous systems are neurotrophins: brain‐derived neurotrophic factor (BDNF), nerve growth factor (NGF) and neurotrophin‐3 and neurotrophin‐4 [3]. BDNF and its receptor Tropomyosin receptor kinase B (TrkB) are expressed on cortical precursor cells and the signalling between those influences the cortical development during embryogenesis [4]. However, it has been shown that the level of circulating BDNF is generally lower in preterm infants than in full‐term infants [5] and that there is a correlation between gestational age and the BDNF levels in cord blood [6]. The latter was also significantly lower in preterm infants who were subsequently diagnosed with intraventricular haemorrhage compared with those without [6].

The premature immune system has a dual role through postnatal transition balancing between tolerance and host defense [7]. The development of the immune system is tightly intertwined with the gut microbiota and their respective metabolites [8, 9]. The colonization with specific bacterial strains differs in the gut of preterm infants and full‐term infants, which might influence the initial immune priming of the premature gut and related immune functions [10, 11]. Bacterial‐derived molecules, like cell wall components of gram‐positive and gram‐negative bacteria or bacterial metabolites, form the link between the gut microbiota and the immune system and their occurrence can shape innate and adaptive immunity [12]. Changes in the gut microbiota are associated with lymphocyte accumulation and differentiation, leading to the production of pro‐ and anti‐inflammatory cytokines and chemokines [8].

Cellular sources of BDNF include various immune cells, such as CD4^+^ T cells, CD8^+^ T cells, B cells, eosinophils [13, 14], vascular endothelial cells [15] and vascular smooth muscle cells [16]. It is tempting to hypothesize that certain bacterial stimuli might influence the BDNF secretion of peripheral blood immune cells in humans. Currently, there is little knowledge about the impact of bacterial stimuli on immune cells leading to the production of BDNF which might indicate a link between the gut, the immune system and the brain. Furthermore, platelets are an additional source of circulating BDNF [17]. BDNF can be released from platelets upon agonist stimulation, for example with thrombin, calcium ionophore or collagen [18]. Platelets show immunomodulatory properties and interact tightly with innate and adaptive immune cells. They can interact with endothelial cells, neutrophils, macrophages, NK cells, B cells and T cells shaping their immune response. Furthermore, platelets impact the immune response by releasing chemokines and cytokines that are stored in their granules [19]. The interaction between platelets and monocytes occurs in different ways: Chemokines and cytokines secreted by platelets can attract or activate monocytes, platelets can be phagocytosed by monocytes, and platelets form aggregates with monocytes [20]. In whole blood, about 45% of monocytes have been observed to form aggregates with platelets, regardless of whether heparinized or citrated blood samples are used [21]. Their interaction influences the phenotype of monocytes [22], the production of chemokines and cytokines such as TNF‐α, IL‐1β, and IL‐8 [23, 24] and the upregulation of β_1_ and β_2_ integrins, which supports the migration of monocytes to inflamed tissue [25].

The aim of this study was to develop a better understanding of the BDNF secretion by peripheral blood immune cells after bacterial stimulation. We observed the response of PBMCs to components of bacterial membranes and metabolites in a time‐ and dose‐dependent manner. As we sought to identify the cellular subset of PBMCs contributing to BDNF levels, we questioned whether immune cells indeed secret BDNF. We looked at the tight interaction between monocytes and platelets, and at the impact of the isolation procedure of monocytes on the concentration of BDNF. We also observed that in vitro differentiated microglia‐like cells do not produce BDNF upon bacterial antigen stimulation. Additionally, we investigated the influence of platelet‐rich and platelet‐poor plasma on the BDNF concentrations in the supernatant of PBMCs to support our hypothesis that platelets are the sole source of BDNF following bacterial stimulation.

## 2 Results

### 2.1 Preterm‐Derived Gut Bacteria Distinctly Modulate Blood Immune Responses

We previously identified that bacterial genera including *Enterococcus*, *Escherichia*, *Klebsiella*, *Staphylococcus*, and *Bifidobacterium* vary in abundance between infants with and without brain injury [9]. In recent years, various immune cell populations have been identified as producers of BDNF, highlighting an intriguing connection between the gut‐immune‐brain axis during the early human development [9, 13, 14]. Since this effect seems to be immunologically mediated, we aimed to investigate the potential immunomodulatory effects of the variously abundant bacterial strains in the preterm gut on BDNF secretion. Therefore, we isolated *Staphylococcus epidermidis* (SE)*, Enterococcus faecalis* (EF)*, Escherichia coli* (EC)*, Klebsiella pneumoniae* (KP), and *Bifidobacterium breve* (BB) from stool samples of our previously recruited PreMiBrain cohort [9] and analyzed the cytokine response utilizing a PBMC stimulation assay in conjunction with T cell activating agents phytohemagglutinin (PHA) or CD3/CD28.

**FIGURE 1:**
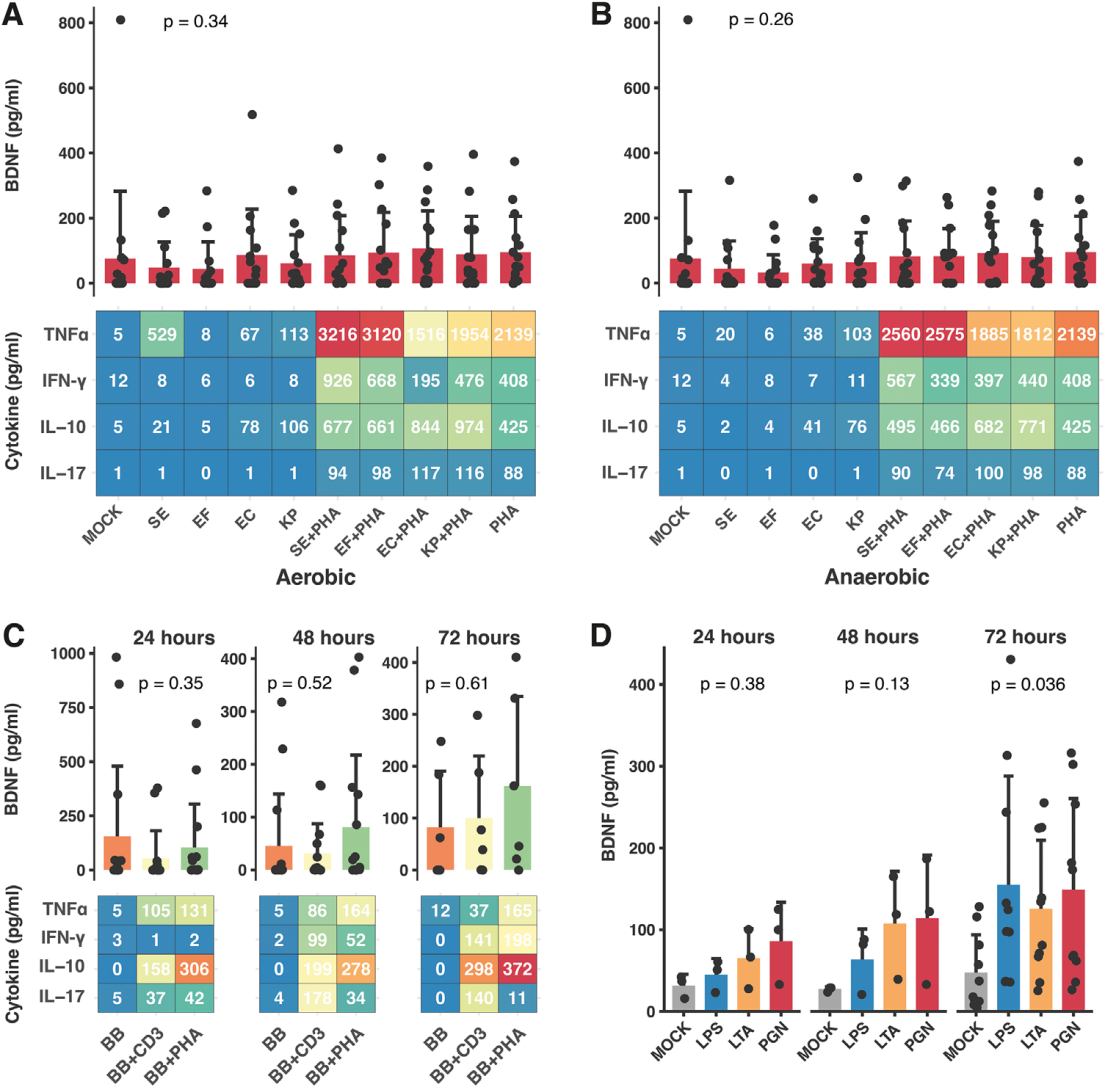
The effect of heat‐killed bacteria and bacterial cell wall components on cytokine and BDNF secretion in human PBMC cultures. The secretion of TNFα, IFN‐γ, IL‐10, and IL‐17 after concomitant stimulation of PBMCs (1 × 10^6^ cells, *n* = 10–15, 10 independent experiments) with phytohemagglutinin (PHA; 5 µg/mL) and heat‐killed *S. epidermidis* (SE), *E. faecalis* (EF), *E. coli* (EC), and *K. pneumoniae* (KP, each 1 × 10^6^) grown under aerobic (A) and anaerobic (B) conditions. Cells were stimulated for 24 h. (C) PBMCs (*n* = 6–15, eight independent experiments) were stimulated with *B. breve* (BB; 1 × 10^6^) as well as PHA (5 µg/mL) or CD3/CD28 (CD; 10 µg/mL) and cytokine and BDNF secretion was analyzed after 24‐, 48‐, and 72 h using ELISA. (D) BDNF concentration over time after stimulation of PBMCs (*n* = 3–10, 4 independent experiments) with LPS (1 µg/mL), LTA (1 µg/mL), or PGN (10 µg/mL). The heatmaps depict the mean concentration (pg/mL) of the indicated cytokines. Bar graphs represent the mean concentration with SD (pg/mL) of BDNF for each corresponding condition. Data were analyzed using the Kruskal–Wallis–Test.

PHA notably enhanced the secretion of TNF‐α, IFN‐γ, IL‐10, and IL‐17. Gram‐positive bacteria, specifically *S. epidermidis* and *E. faecalis*, predominantly elevated TNF‐α and IFN‐γ levels, whereas gram‐negative *E. coli* and *K. pneumoniae* notably influenced IL‐10 and IL‐17 production. Oxygen conditions during bacterial cultivation did not significantly alter cytokine secretion. The mean BDNF secretion varied extensively, generally increasing upon PHA stimulation of PBMCs (Figure 1A/B). However, we also observed BDNF release in some unstimulated conditions (referred to as MOCK). Summarized cytokine data are depicted in Figures S1 and S2.

*B. breve* alone or combined with CD3/CD28 or PHA revealed no consistent cytokine response pattern. Notably, BDNF levels remained low at 24‐ and 48 h post‐incubation but increased after 72 h of concomitant stimulation (Figure 1C).

To standardize the assessment of Gram‐positive and Gram‐negative bacterial effects on BDNF secretion, cell wall components lipopolysaccharide (LPS), lipoteichoic acid (LTA), and peptidoglycan (PGN) were applied to PBMCs. All ligands increased BDNF levels in supernatants compared with controls, with the highest concentrations observed after 72 h of incubation (Figure 1D).

Overall, heat‐killed bacteria derived from the preterm gut as well as bacterial cell wall components exhibited immunomodulatory properties in healthy adult PBMCs. Furthermore, bacterial stimulation partly induced a BDNF response in adult PBMCs.

### 2.2 Short‐Chain Fatty Acids Influence BDNF Production of PBMC Cultures

We were able to demonstrate that bacterial antigens can induce BDNF secretion in human PBMC cultures. In addition, microbial‐derived metabolites have an impact on the developing immunity of infants [26]. We recently described differences in the concentration of SCFAs in the stool of extremely premature infants with or without brain injuries, which led us to look closer at the effect of sodium‐butyrate, sodium‐acetate and sodium‐propionate on the secretion of BDNF by PBMCs [9].

**FIGURE 2:**
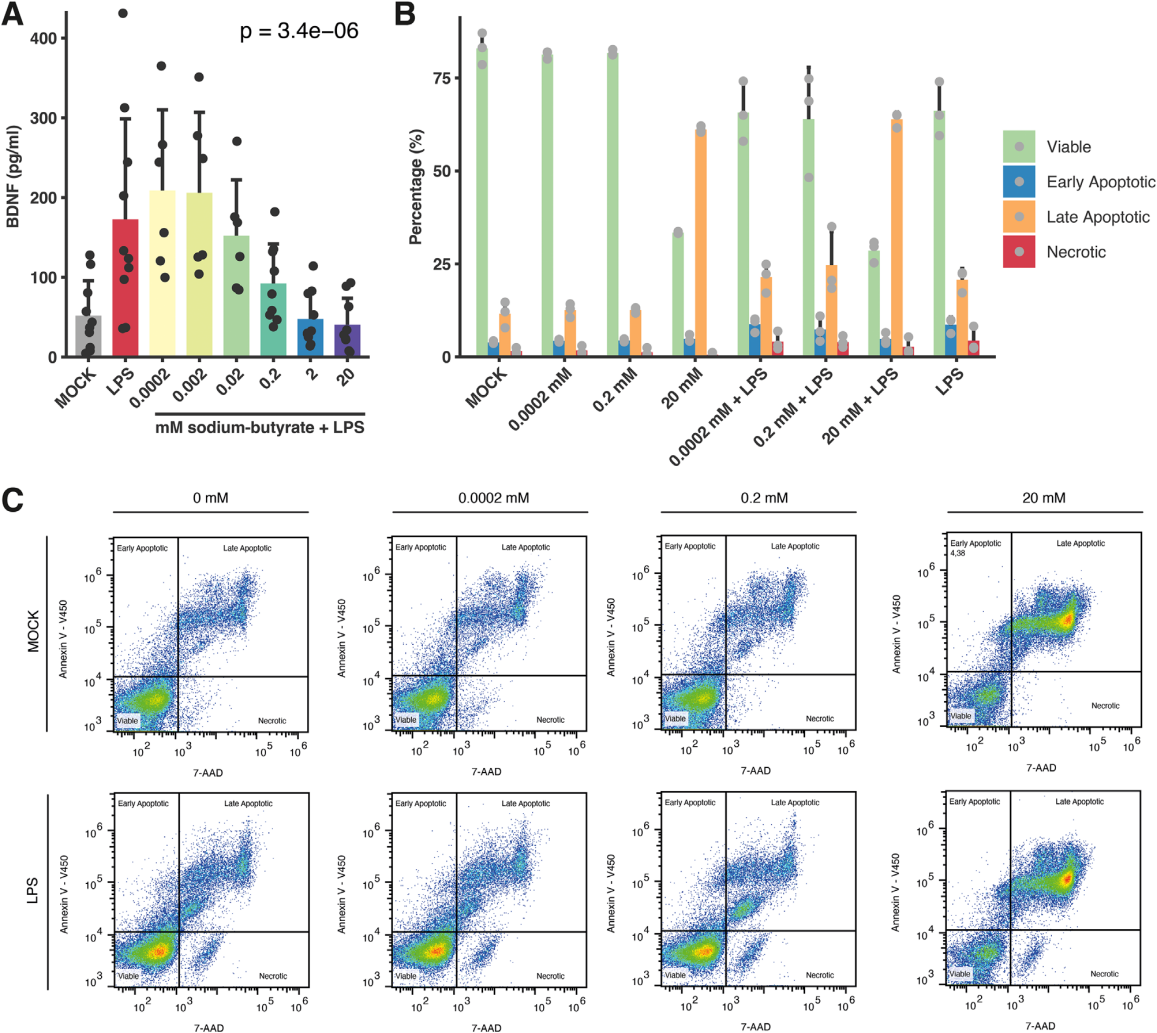
Short‐chain fatty acids (SCFAs) influence the BDNF production of stimulated PBMCs in a dose‐dependent manner. (A) Dose‐kinetic (mM range) of concomitant stimulation of PBMCs (*n* = 6–10, four independent experiments) with sodium‐butyrate as well as LPS (1 µg/mL). The BDNF concentration was determined after 72 h of cell culture stimulation using ELISA. (B) The effect of high concentrations of sodium‐butyrate on the viability of PBMCs (*n* = 3, one independent experiment) with or without LPS (1 µg/mL) stimulation as assessed via flow cytometry. (C) Representative flow cytometry dot plots of the apoptosis assay using Annexin V and 7‐AAD staining. Bar graphs represent the mean with SD. ANOVA was used for statistical analysis.

While the sole administration of SCFAs did not modify BDNF levels, simultaneous stimulation with bacterial cell wall components significantly enhanced BDNF secretion, surpassing that of the unstimulated control. However, this co‐stimulation did not achieve BDNF concentrations higher than those elicited by bacterial cell wall components alone. Intriguingly, we noted a tendency for higher SCFA concentrations to inversely correlate with BDNF levels in the supernatant (Figure 2A; Figure S3). Among the SCFAs we investigated, sodium‐butyrate exhibited the most significant effect, prompting us to explore concentrations that reflect physiological in vivo conditions. Butyrate levels vary across the gut, peaking in the caecum (26.1 ± 3.8 mmol/kg) and decreasing towards the colon. In the bloodstream, levels drop to around 15.1 ± 3.5 µM and further decline to 0.5 ± 0.1 µM posthepatic absorption. This concentration gradient, from gut to liver, underscores the physiological significance of butyrate and informs our selection of experimental concentrations to reflect these natural conditions [27].

Again, bacterial stimulation markedly augmented BDNF levels, and synergistic co‐stimulation with bacterial cell wall components and sodium‐butyrate at minimal concentrations (0.0002 and 0.002 mM) showed a trend to amplify BDNF secretion (Figure 2A; Figure S3). Conversely, higher concentrations of sodium‐butyrate diminished BDNF secretion, potentially due to cytotoxic effects. Notably, sodium butyrate has been shown to influence cancer cell growth dynamics, with concentrations ranging from 1–2 mM affecting cell proliferation and 8–16 mM inhibiting tongue cancer cell growth by up to 80% [28]. This cytotoxicity was corroborated in our study utilizing a flow cytometry‐based apoptosis assay, which revealed a significant increase in apoptosis at a 20 mM concentration. This effect was exacerbated by the addition of LPS (Figure 2B,C). These findings suggest that apoptosis induced by high concentrations of sodium‐butyrate may account for the reduced BDNF levels observed in samples treated with 20 mM of the compound. However, the observed decrease of BDNF at lower concentrations (0.0002 and 2 mM) without a corresponding increase in apoptosis might suggest the involvement of alternative regulatory mechanisms. SCFA acts via inhibition of histone deacetylase (HDAC) and can alter gene expression by affecting chromatin structure and accessibility. It is possible that HDAC inhibition at these lower concentrations affects the transcriptional regulation of genes involved in BDNF synthesis and secretion in PBMCs [29, 30].

### 2.3 T Cells and Monocytes Do Not Serve as the Cellular Origin of BDNF

In the next step, we sought to pinpoint the major cellular source of BDNF secretion. In previous studies, T cells, B cells, and monocytes have been described as a source of circulating BDNF upon stimulation [13, 31]. Our initial experiments suggested that T cells might contribute significantly to the BDNF production of PBMCs. To characterize the subpopulation of PBMCs secreting BDNF, we utilized various intracellular staining protocols to assess BDNF levels in PBMCs.

**FIGURE 3:**
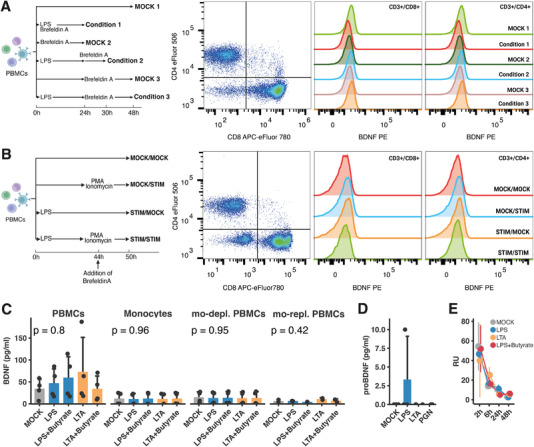
Approaches to identify PBMCs as the cellular origin of BDNF. (A) Stimulation scheme for the intracellular detection of BDNF in CD3^+^/CD4^+^ and CD3^+^/CD8^+^ cells after stimulation with LPS (1 µg/mL) using flow cytometry. A representative flow cytometry dot plot as well as a histogram of the different conditions are shown for one experiment (total *n* = 2). (B) Stimulation scheme for the intracellular detection of BDNF in CD3^+^/CD4^+^ and CD3^+^/CD8^+^ cells after stimulation with LPS (1 µg/mL), PMA (50 ng/mL), and Ionomycin (1 µg/mL). A representative flow cytometry dot plot as well as a histogram of the different conditions are shown for one experiment (total *n* = 2). (C) BDNF concentration in cellular fractions after the magnetic isolation of monocytes as assessed by ELISA (*n* = 4, four independent experiments). Bar graphs represent the mean concentration with SD (pg/ml) of BDNF for each corresponding condition. Cells were stimulated with LPS (1 µg/mL) or LTA (1 µg/mL) with or without sodium‐butyrate (2 mM) for 72 h. (D) proBDNF concentration after 72 h of stimulation of PBMCs (*n* = 3, one independent experiment) with LPS (1 µg/mL), LTA (1 µg/mL), or PGN (10 µg/mL). proBDNF concentrations were determined by ELISA. (E) The temporal change of BDNF mRNA expression (*n* = 3, one independent experiment) over the course of 48 h in the unstimulated control and stimulated samples: LPS (1 µg/mL), LTA (1 µg/mL), LPS (1 µg/mL) and sodium‐butyrate (2 mM). Data were analyzed using the Kruskal–Wallis test.

In the first approach, we used Brefeldin A for the accumulation of proteins in the endoplasmatic reticulum and LPS (1 µg/mL) as a stimulant at different time points. Figure 3A depicts the experimental stimulation setup. In all conditions, we were unable to detect a BDNF signal in T cell populations. In the second approach, PBMCs were again stimulated with LPS (1 µg/mL), and PMA (50 ng/mL) and ionomycin (1 µg/mL) were added to increase the cytokine production (Figure 3B). Again, we were not able to detect intracellular BDNF in T cell subsets.

Given the absence of BDNF production in T cells, we postulated that monocytes might be the primary contributors of BDNF in our cell culture experiments. Prior studies have shown that monocytes can release BDNF following stimulation with CD14‐coated magnetic beads [13] and in response to bacterial interactions [32]. Although we stained monocytes for our flow cytometry experiments, strong downregulation of CD14 made it impossible to sufficiently distinguish the monocyte population (Figure S4). Therefore, our alternative strategy was to isolate monocytes using magnetic bead separation and conduct parallel cultures of three different cell groups: PBMCs, isolated monocytes, and monocyte‐depleted PBMCs. As depicted in Figure 3C, BDNF measurements indicated elevated levels exclusively in the PBMC samples, with no detectable BDNF in either the isolated monocyte group, the monocyte‐depleted or monocyte‐replenished PBMCs. Furthermore, we could not detect proBDNF secretion after bacterial antigen stimulation (Figure 3D).

To further corroborate these findings, we assessed the temporal dynamics of BDNF mRNA expression in PBMCs, measuring mRNA levels at 2‐, 8‐, 24‐, and 48 h poststimulation. BDNF mRNA expression remained consistent across all conditions, with a parallel decline observed over time. Notably, stimulation with LPS (1 µg/mL), LTA (1 µg/mL), and a combination of LPS (1 µg/mL) with sodium butyrate (2 mM) failed to elicit an increase in BDNF mRNA levels in PBMCs, further supporting our initial observations (Figure 3E).

In conclusion, our efforts to identify a specific cellular origin for BDNF within human PBMCs were unsuccessful. Despite observations that stimulation with bacterial cell wall components can enhance BDNF concentrations in cell culture supernatants, the effects were abolished when monocytes were isolated from PBMCs, indicating the complexity of BDNF production dynamics in this context.

### 2.4 Magnetic Separation Reduces the Number of Monocyte‐Platelet Aggregates

**FIGURE 4:**
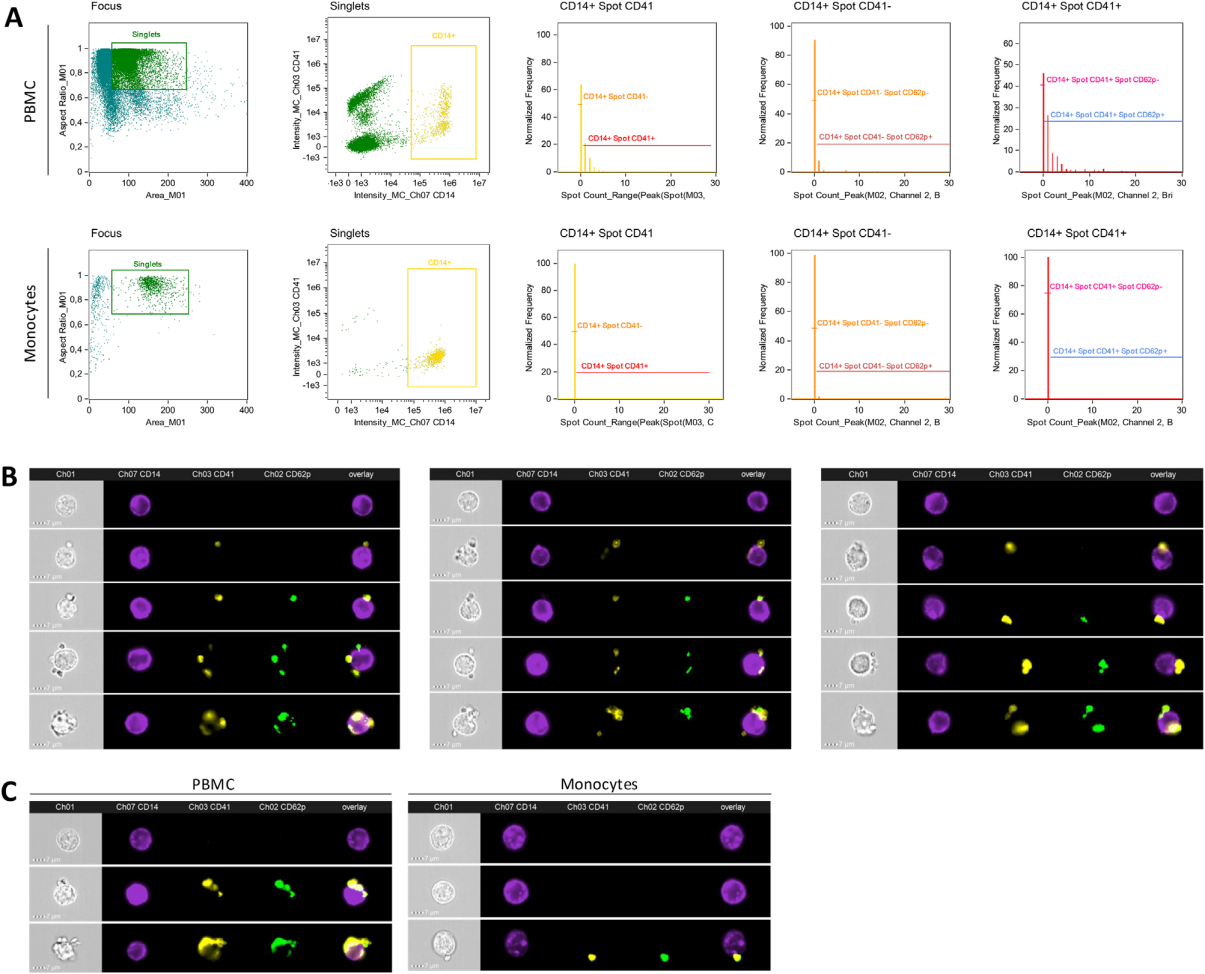
Imaging flow cytometry of monocyte‐platelet aggregates. (A) Spot analysis of PBMCs (upper panel) and CD14^+^ magnetically isolated monocytes for CD41^+^ and CD62p^+^ platelets. A representative gating strategy of one sample is shown from a total of *n* = 3. (B) Imaging flow cytometry reveals the spatial interaction between monocytes and platelets in PBMCs. Data from two independent experiments are shown. (C) Visualisation of the difference between monocyte‐platelet aggregates in PBMCs and isolated monocytes. Used markers: CD14^+^ (BV421) is used for monocytes, CD41^+^ (PE‐Cy7) is used for platelets, and CD62^+^ (FITC) is used for activated platelets.

**FIGURE 5:**
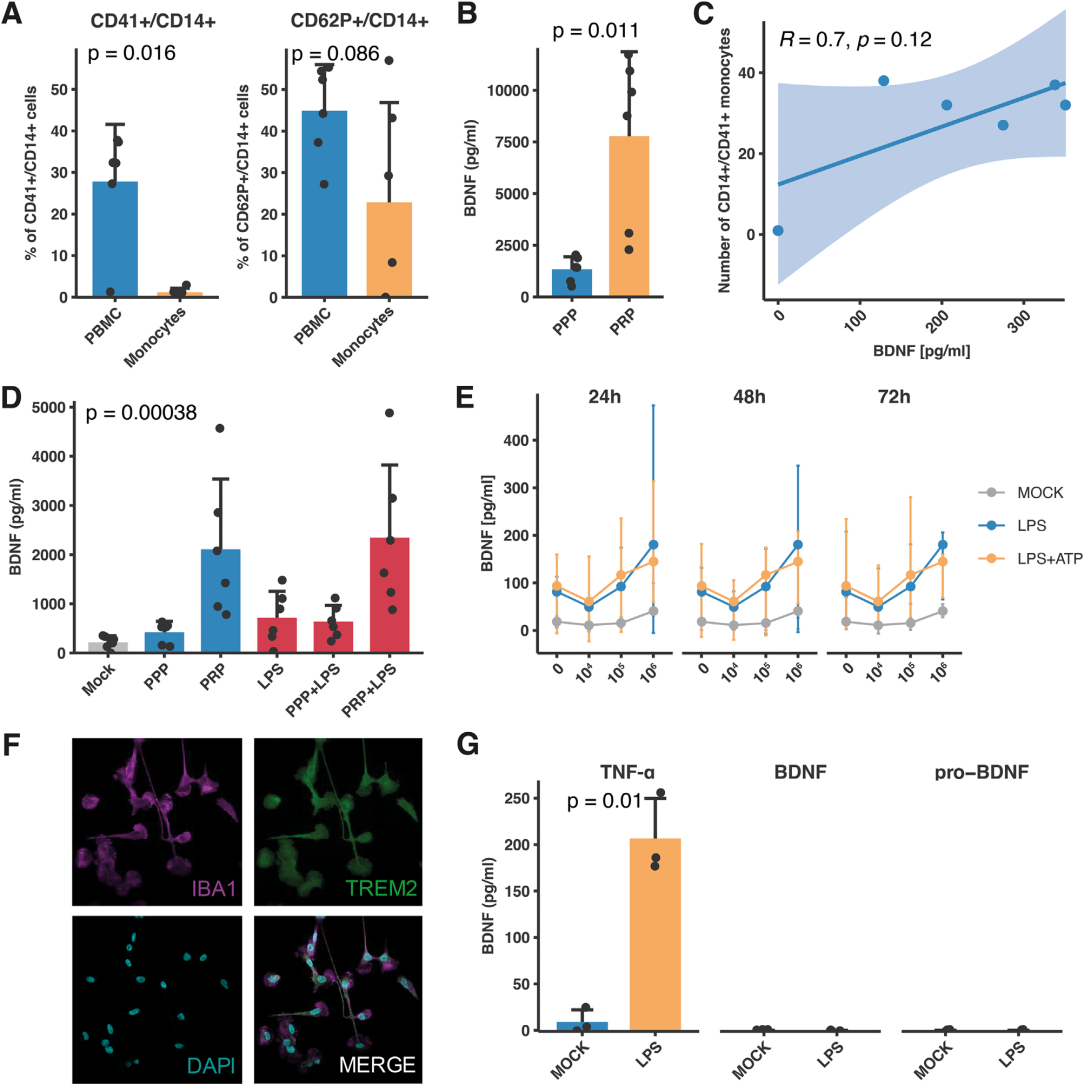
The influence of platelets on the BDNF concentration in the supernatant of PBMC cultures. (A) Result of the imaging flow cytometry spot analysis (*n* = 6, three independent experiments) illustrating the percentage of CD41^+^ monocytes (left graph). The right graph depicts the percentage of CD41^+^/CD62P monocytes. (B) BDNF content in freeze/thawed platelet‐poor plasma (PPP) and platelet‐rich plasma (PRP), as analyzed by ELISA (*n* = 6, three independent experiments). (C) The number of CD14^+^/CD41^+^ monocytes, as assessed by imaging flow cytometry, correlates positively with the BDNF concentration of the corresponding cell culture supernatant. (D) The changes in BDNF concentration by the addition of PPP or PRP. Cells were stimulated for 72 h, and the supernatant was analyzed using ELISA. LPS was further used as a stimulant at a concentration of 1 µg/mL. (E) PBMCs were stimulated with LPS (1 µg/mL) as well as in addition with ATP (0.5 mM) for 72 h. Additionally, a defined number of isolated platelets were added to the cell culture and BDNF was assessed using ELISA (*n* = 3, two independent experiments). (F) Human microglia were generated in vitro from the human embryonic stem cell line WA09. Immunocytochemistry confirmed the expression of microglial markers IBA1 and TREM2 in the MGLs. (G) Microglia (*n* = 3; three independent experiments) were stimulated for 24 h with LPS at a concentration of 100 ng/mL. The secretion levels of TNF‐α, BDNF, and pro‐BDNF were measured using ELISA. A *t*‐test or Mann–Whitney *U*‐test was utilized to assess the statistical significance between two conditions. For comparison of multiple means, ANOVA was used. Correlation analysis was conducted using Pearson statistics.

We could demonstrate that BDNF levels in the supernatant of PBMCs increase upon stimulation with bacterial antigens, however, we were unable to identify the specific cellular origin. Upon further investigation of blood components with immunological properties, platelets have been shown to interact closely with immune cells, especially with monocytes [33, 34]. Recently, it has been described that circulating platelets store BDNF in α‐granules which can be released during activation [17]. Therefore, we hypothesized that monocyte‐platelet aggregates could be a potential source for BDNF secretion in PBMC cultures. Imaging flow cytometry can provide a detailed view of cellular interactions, particularly in the identification and analysis of monocytes and platelets within PBMCs and CD14^+^ magnetically isolated monocyte populations. Utilizing CD14 as a monocyte surface marker, CD41 to identify platelets and CD62p for activated platelets, we observed distinct cellular dynamics after the magnetic cell separation of monocytes. As illustrated in Figure 4A, the spot analysis revealed differences in CD14^+^ monocytes coupled with CD41^+^ platelets in PBMCs and magnetically separated monocytes: In PBMCs, we identified aggregates consisting of CD14^+^ monocytes and CD41^+^ platelets. However, following magnetic isolation, these aggregates were scarcely observed, indicating a significant decrease in CD14^+^/CD41^+^ cells after the magnetic separation of monocytes (Figure 5A). This suggests that the magnetic separation of monocytes effectively disrupts monocyte‐platelet aggregates.

Further examination of PBMCs revealed CD14^+^ cell aggregates exhibiting both positive and negative signals for CD41, with CD14^+^/CD41^+^ cells showing an enhanced signal for CD62p, indicative of activated platelets. Interestingly, aggregates displaying CD62p positivity were not exclusively associated with CD41^+^ cells, suggesting varied activation states and interactions within the cellular milieu. Notably, CD62p^+^ events were nearly absent in isolated monocyte populations, underscoring the impact of magnetic separation on cellular interactions.

The spatial dynamics between monocytes and platelets were further elucidated through imaging analyses. Figure 4B presents bright field images of monocyte‐platelet aggregates from three different probands, clearly showing platelets in direct contact with monocytes. The structural analysis of these aggregates revealed diversity in their composition—ranging from monocytes attached to a single platelet (either activated or inactivated) to those connected to multiple platelets, with variations in the proximity of the platelets to one another. This comprehensive analysis underscores the interplay between monocytes and platelets within the immune response, emphasizing the significance of cellular context and the impact of methodology on observed interactions.

Imaging analysis further visualized the effect of the magnetic separation on the aggregates. For direct comparison, images from PBMCs and isolated monocytes from one proband are depicted in Figure 4C. Magnetically isolated monocytes were rarely attached to platelets, and when they were, it was typically only to a single one. In contrast, monocytes in PBMC samples were often attached to either a single platelet or to multiple platelets, forming platelet‐loaded aggregates.

### 2.5 Platelet‐Rich Plasma Increases BDNF Levels in Stimulated PBMC Cultures

We ascertained that cultures of PBMCs may harbour a considerable number of monocyte‐platelet aggregates, which are disrupted by the process of positive magnetic separation. Our prior investigations have clearly demonstrated that these aggregates constitute the primary source of BDNF observed in PBMC cultures after bacterial stimulation. Platelets store signalling molecules, including BDNF, which are sequestered within α‐granules and the cytosolic compartment. The release of BDNF is precipitated by exposure to agents such as thrombin, collagen, calcium ionophores, and shear stress [17, 18, 35].

Next, we sought to identify how the addition of platelets might influence the BDNF response in PBMC cultures. Our findings demonstrated that platelet‐rich plasma (PRP), derived from blood collected in citrate tubes, contains significantly higher levels of BDNF compared with platelet‐poor plasma (PPP, Figure 5B). PRP and PPP were frozen and thawed prior to analysis to measure the overall content of BDNF within platelets. Furthermore, we found a positive correlation between CD14^+^/CD41^+^ aggregates as assessed by imaging flow cytometry and the concentration of BDNF with a correlation coefficient of 0.7, which suggests that the number of CD14^+^/CD41^+^ events might contribute to the concentration of BDNF (Figure 5C).

Consequently, we added both the PPP and PRP to PBMCs obtained from heparinized blood. While PPP had a marginal impact on the BDNF concentration, the addition of PRP enhanced the BDNF concentration in unstimulated (MOCK) and LPS‐stimulated conditions. However, there was no difference in BDNF concentrations between unstimulated and LPS‐stimulated PBMCs with added PRP, suggesting that the effect is not amplified by the higher platelet count in citrate‐based PRP (Figure 5D). However, the addition of PPP clearly enhanced the overall BDNF concentrations compared with LPS stimulation alone as depicted in Figures 1D and 5D.

To determine whether the observed effects were attributable to heparin, we explored the impact of platelet concentration on BDNF levels by supplementing PBMCs with a known quantity of platelets from heparinized blood. We then measured BDNF concentrations in both unstimulated controls and samples stimulated with LPS (1 µg/mL) alone or in combination with ATP (0.5 mM). The rationale for using ATP was to amplify the LPS signalling as well as further activation of platelets. Our findings, presented in Figure 5E, revealed a consistent trend of increasing BDNF concentrations in correlation with platelet counts across all conditions, including both unstimulated and stimulated samples. These observations collectively imply that platelets are the predominant contributors to BDNF secretion in human blood circulatory cells.

### 2.6 Microglia Do Not Produce BDNF after In Vitro LPS Stimulation

Microglia are pivotal to immune surveillance in the central nervous system, functioning as resident phagocytes that continuously monitor the brain's microenvironment and respond to pathological challenges. Beyond their immune roles, microglia are essential for neural development, synaptic formation and plasticity, the maturation of neural networks, and the refinement of neuronal circuits through synaptic pruning. Microglial dysfunction has been associated with a broad spectrum of brain disorders throughout the human lifespan, from prematurity‐related brain injuries in infants to neurodegenerative and psychiatric conditions in the elderly. While microglia and peripheral blood monocytes originate from distinct developmental lineages, they show similar functions during inflammation. Therefore, we investigated whether human embryonic stem cell‐derived microglia secrete BDNF in response to LPS stimulation. Using the differentiation protocol described by Haenseler et al. [36], our differentiated microglia displayed the expected phenotype (Figure 5F; Figure S4). Upon stimulation with LPS, microglia exhibited a significant secretion of TNF‐α; however, they did not secrete BDNF or pro‐BDNF into the cell culture supernatant (Figure 5G). Our findings suggest that BDNF secretion in human immune cells, even in those which are residing in highly specialized immunological niches, may be regulated by a more complex network of signalling pathways than a single bacterial stimulus alone can trigger.

## 3 Discussion

Following the concept that immune cells secrete BDNF in the human body [13], we aimed to gain a deeper understanding of BDNF secretion by peripheral blood immune cells following exposure to bacterial antigens. We showed that the BDNF level in PBMC culture is impacted by bacterial antigen stimulation, but we were not able to pinpoint T cells or monocytes as the primary source of BDNF [13]. Since platelets store BDNF [18, 35] and closely interact with immune cells [37], we visualized the spatial interaction between monocytes and platelets forming aggregates. We found that the number of these aggregates was significantly reduced after magnetic‐bead separation, which corresponded with a concomitant decrease in BDNF levels in the culture supernatant. Conversely, we found that the BDNF concentration increased with a rising number of platelets. Even in highly specialized cells such as microglia‐like cells, we could not detect BDNF secretion upon LPS activation. Based on our experiments, we concluded that platelets are the sole source of BDNF in PBMC cultures and that their quantity significantly influences the BDNF concentration following bacterial antigen exposure.

Our findings show that heat‐killed bacteria and defined bacterial cell wall components modulate PBMC responses, particularly affecting BDNF secretion. Heat‐killed bacteria produced variable BDNF concentrations, while standardized cell wall components (LPS, LTA, PGN) induced a time‐dependent increase in BDNF, peaking at 72 h compared with unstimulated controls. The effect of LPS on BDNF secretion in PBMCs aligns with the studies by Shen et al. [32] and Enstrom et al. [38], using similar culture approaches. As SCFAs influence the immune system [39] and differ in preterm infants with and without brain injury [9], we examined their effect on BDNF secretion in PBMCs stimulated with bacterial components. SCFAs (2 mM) alone did not alter BDNF levels, consistent with Asarat et al.’s findings that similar SCFA concentrations did not affect various cytokines [40]. Co‐stimulation with bacterial antigens and SCFAs increased BDNF compared with controls but never surpassed antigen‐only levels. Increasing SCFA concentrations reduced BDNF, with butyrate showing the strongest effect. Using physiological butyrate concentrations, low doses enhanced BDNF, while higher concentrations decreased it, likely due to cytotoxicity [41]. Recent work suggests SCFAs may also indirectly influence platelet function, warranting future studies on SCFA‐mediated platelet‐derived BDNF [42].

Kerschensteiner et al. [13] showed that T cells are capable of secreting BDNF following antigen‐specific stimulation or activation via PHA after 72 h. We observed a similar trend in our stimulation assays with CD3/CD28 as well as PHA. Nevertheless, we were interested in T cells are a potential source after bacterial antigen encounter. We did not detect intracellular BDNF in CD4+ and CD8^+^ T cells and could therefore not define them as a cellular source without a concomitant T cell stimulant. However, although platelet‐lymphocyte aggregates have been described previously at a lower frequency compared with monocyte‐platelet aggregates, they may contribute to the release of BDNF in peripheral blood as well [43]. Furthermore, we did not detect BDNF in the supernatant of isolated monocytes, ruling out the possibility that secretion is based on cellular or secretory interactions. Interestingly, the analysis of the mRNA of PBMCs showed a decrease of mRNA over time, suggesting that BDNF is not transcribed during the stimulation period. Therefore, we concluded that the secretion of BDNF is influenced by other factors which needed to be defined.

Immune cells and platelets interact in various ways [19], thereby modulating the immune response [37]. We demonstrated spatial dynamics between monocytes and platelets using imaging flow cytometry. Platelets are capable of forming aggregates of different compositions and activation levels on the surface of monocytes, as indicated by the expression of CD62p (Figure 4). The number of monocyte‐platelet aggregates was significantly lower in magnetically‐isolated monocytes than in PBMCs. Based on this observation and the lack of BDNF in magnetically isolated monocytes, we concluded that platelets are a critical player in the BDNF secretion of PBMCs after bacterial antigen stimulation. This conclusion is further supported by our finding that only BDNF—and not proBDNF—is secreted after bacterial stimulation, implying platelet activation [44]. This contrasts with the findings of Kerschensteiner et al. [13], where BDNF secretion was identified after isolation with CD14^+^ microbeads alone. However, Hawwari et al. [45] recently showed that platelets are essential players in monocyte activation and induction of pro‐inflammatory responses. This hypothesis is further supported by the positive correlation between the number of CD14^+^/CD41^+^ events and the BDNF concentration in the cell culture (Figure 5C), as well as by the finding of significantly higher BDNF concentrations in PRP compared with PPP derived from citrate tube blood samples (Figure 5B). The same effect was observed when PRP and PPP were added to PBMCs derived from heparinized blood (Figure 5D). However, the additional stimulation with LPS (1 µg/mL) did not further enhance the BDNF concentration in PRP‐added samples. We also examined the effect of a defined platelet count from heparinized blood added to PBMCs and observed that, once again, the higher number of platelets resulted in an elevated concentration of BDNF.

We concluded that platelets are the sole source of secreted BDNF in bacterial antigen‐stimulated PBMCs, contributing to the variability in BDNF concentrations observed in our experiments and potentially throughout the literature (Table 1). Differences in the treatment of blood samples until the measurement of BDNF regarding isolation procedure, collection tubes, washing procedures and the efficacy of magnetic separation might influence the number of platelets in the final sample. Platelet isolation is usually performed with 150–200*g*, which is typically aimed at minimizing the loss of large platelets. The higher centrifugation speed of the PBMC protocol might contribute to the inadvertent pelleting of significant subpopulations, potentially leading to an observed higher concentration of platelets. This variation in centrifugation conditions and its impact on platelet yield and purity should be carefully considered. In Table 1, we compared our experimental setup of the isolation and stimulation of monocytes with those used in selected studies [13, 31, 32, 46, 47, 48]. The focus of previous research has primarily been on comparing BDNF concentrations between healthy individuals and those with medical conditions. However, the concentration of BDNF in the supernatant of monocytes varies widely, ranging from 20 to 400 pg/mL, which is about a 20‐fold difference. In our experiments, we only measured 0–20 pg/mL. This discrepancy raises the question of which factors contribute to such a wide range of BDNF concentrations. To our knowledge, the influence of platelets and the corresponding adaptations to the experimental setup has not been adequately considered in studies examining PBMCs or monocytes as the cellular source of BDNF.

**TABLE 1:** Comparison of the experimental set‐up of the isolation and stimulation of monocytes.

Author	M. Kerschensteiner [13]	B. Rost [31]	O. Schulte‐Herbrüggen [49]	D. Azoulay [50]	S. Yoshimura [48]	Wei‐Yun Shen [32]	Our experiment
Year	1999	2005	2005	2008	2010	2019	2023
Probands	Not specified	Healthy Controls, Patients with allergic asthma	Healthy probands	Healthy Controls and MS patients (untreated)	Healthy controls and MS patients (untreated and treated)	Healthy controls and Patients with Stanford type‐A aortic dissection	Healthy probands
Anticoagulant	Not specified	Heparin	Not specified	Heparin	EDTA tubes	Not specified	Heparin
PBMC isolation	Ficoll‐Paque	Ficoll‐Paque	Lymphoprep	Ficoll‐Paque	Lymphoprep	Lymphoprep	Ficoll‐Paque
Isolation of monocytes	Immuno‐magnetic beads (Dynal)	Magnetic‐activated cell sorting (Miltenyi Biotech)	Magnetic‐activated cell sorting (Miltenyi Biotech)	Magnetic‐activated cell sorting (Miltenyi Biotech)	Magnetic‐activated cell sorting (Miltenyi Biotech)	Magnetic‐activated cell sorting (Miltenyi Biotech)	Magnetic‐activated cell sorting (Miltenyi Biotech)
Stimulant	no stimulants	LPS: 10, 1, 0.1 µg/mL	Unstimulated control	No stimulants	No stimulants	LPS 1 µg/mL	unstimulated control LPS 1 µg/mL
Time of incubation	72 h	24 and 48 h	24 h	24 h	48 h	72 h	72 h
Cell concentration	not specified	10^5^ cells/well	4 × 10^5^ cells/well	not specified	1 × 10^5^ cells/well	2 × 10^6^ cells/mL	2×10^5^ cells/mL
Medium	RPMI 1640 + 5% FCS	RPMI 1640 + 10%FCS + 5% penicillin/streptomycin	serum‐free AIM‐V with 1% L‐glutamine, 1.25 µg/mL fungizone	RPMI 1640 medium + 4 mM L‐glutamine + 25 mM Hepes buffer + penicillin 50 units/mL + streptomycin 50 µg/mL	X‐Vivo 15	IMDM medium +10% FBS + 2 mM L‐glutamine + 100 U/mL penicillin + 0.1 mg/mL streptomycin	RPMI Medium 1640 (1X) + GlutaMAX^TM^‐I + 10% FBS
Detection	ELISA: antibodies from Promega; Research Diagnostics, Inc.; Dianova	BDNF ELISA ‐R&D Systems	BDNF ELISA Kit from Promega	DuoSet ELISA Development System (R&D System)	ELISA in situ (Promega)	m‐BDNF‐specific sandwich ELISA by Dr. Zhou [51]	DuoSet ELISA Development System (R&D SYSTEM)
Measured BDNF [6]	300	20–30 (unstimulated and stimulated samples of healthy controls)	50 (unstimulated)	Healthy control: 382.6±277.9;	Healthy control: 350	Healthy controls: 400 (unstimulated) 600 (stimulated)	0–20

Abbreviations: BDNF—brain‐derived neurotrophic factor; ELISA—enzyme‐linked immunosorbent assay; FBS—fetal bovine serum; FCS—fetal calve serum; LPS—lipopolysaccharide; MS—multiple sclerosis.

Given our suggestion that platelets may play a crucial role in determining BDNF concentrations in the supernatant of immune cells, it might be important to carefully select the anticoagulant used for blood collection. The anticoagulants mentioned in the literature are either heparin or EDTA, or they are not specified. Heparin is the standard anticoagulant for immunological studies, however, it might not be the best choice when investigating the influence of platelets, as it can affect cell properties such as platelet activation [52, 53]. Within the literature, the PBMC isolation techniques as well as the monocyte isolation protocols rely on the same principles. We have shown that the number of monocyte‐platelet aggregates decreases significantly after the sorting process, which might be another important factor to consider when evaluating BDNF concentrations in the supernatant of monocytes. The cell concentration as well as the cell culture medium and its additives vary throughout the studies. Additionally, the detection methods differed, which might further contribute to the wide range of BDNF concentrations reported.

Taken together, the experimental setup for measuring BDNF in the supernatant of immune cells varies greatly, which appears to influence the measured BDNF concentrations. The experimental setup has traditionally been optimized, for immune cells which, with our current understanding, may have led to an underestimation of the influence of platelets. To generate more consistent data, it may be crucial to establish a protocol specifically adapted to the challenges of working with platelets. Assuming that the cause of altered BDNF concentrations in individuals with certain conditions such as multiple sclerosis [48 ^,^ 50], allergies [31], and autism [38] are due to platelet malfunctions rather than immune cell abnormalities would necessitate further, more detailed research into the role and mechanisms of platelets in these conditions.

### 3.1 Data Limitations and Perspectives

Although we explored various experimental approaches to detect BDNF secretion in PBMCs, we encountered several methodological limitations. Further investigations are required to elucidate the direct and indirect effects of bacterial antigen encounters on platelet activation and their subsequent impact on BDNF secretion. Additionally, refining our flow cytometry method may allow for more precise identification of the CD41^+^ monocyte population, aiming to detect intracellular BDNF within potential monocyte‐platelet aggregates. Moreover, Western blot analyses could further validate the presence or absence of BDNF in these CD41^+^ monocyte samples.

## 4 Methods

### 4.1 Ethical Approval and Study Participants

In this study, 17 healthy adults aged between 21 to 33 years were included. The consent to participate in the study was obtained by the volunteers prior to study inclusion. The study was approved by the ethics committee of the Medical University of Vienna (ethics application number 1415/2019).

### 4.2 Isolation of Bacteria from Stool Samples

Premature infant stool samples were diluted in 1x DBPS^−/−^ (Gibco) and were used as the inoculum for plating the stool on the respective media: *Bifidobacterium breve* and *Staphylococcus epidermis* on sBHI (solid brain heart infusion media), *Enterococcus faecalis*, *Klebsiella pneumonia*, and *Escherichia Coli* on PYG (peptone yeast extract glucose medium). The bacteria were incubated for 24 h under anoxic conditions in a COY vinyl type A anaerobic chamber (Coy Laboratory Products, 80% N_2_, 15% CO_2_, 5% H_2_). Singular morphotypes were obtained by transferring morphologically different colonies on fresh plates and incubating for another 24 h. This process was repeated to ensure purity. Additionally, *Staphylococcus epidermis, Enterococcus faecalis, Klebsiella pneumonia*, and *Escherichia Coli* were grown under aerobic conditions at 37°C and 5% CO_2_. Heat‐killed bacteria were generated by autoclaving the bacteria for 15 min at 121°C. The heat‐killed cultures were centrifuged for 5 min at 14000 rpm and the pellet was resuspended in 1× DBPS^−/−^ (Gibco). Bacteria were fixed using ethanol for Gram‐positive bacteria and paraformaldehyde for Gram‐negative bacteria. After fixation, the bacteria were deposited onto a 0.2 µm filter. This filter was then incubated in a DAPI solution (10 µg/mL) for 5 min to stain the nucleic acids. Following staining, the filters were mounted on microscopy slides using Citifluor (EMS, Hatfield, PA, USA) and covered with a coverslip. Cell enumeration was performed using a 100× oil immersion objective on an Axioplan 2 imaging fluorescence microscope (Zeiss, Oberkochen, Germany).

### 4.3 PBMC Isolation

For the isolation of PBMCs, venous blood was drawn into heparinized blood collection tubes and diluted 1:2 with DBPS^−/−^ (Gibco). For the separation of PBMCs, Leucosep tubes were filled with Ficoll (Cytiva) according to the manufacturers’ instructions and centrifuged for 1 min at 1000*g* at room temperature. The diluted blood was added to the Leucosep tubes which were further centrifuged for 15 min at 300*g* at room temperature. The cell layer containing PBMCs was isolated, and cells were washed two times for 10 min at 300*g* at room temperature. Viable cells were counted with the Luna‐FL Dual Fluorescence Cell Counter (logos biosystems). The cell concentration in the sample was adjusted by resuspending the cell pellet in the according volume of full culture medium (RPMI Medium 1640 (Thermo Fisher) + 10% FBS (Thermo Fisher)).

### 4.4 Stimulation of PBMCs with Heat Killed Bacteria

PBMCs were seeded at a concentration of 1 × 10^6^ cells/mL cell culture medium (RPMI Medium 1640 (Thermo Fisher) + 10% FBS (Thermo Fisher)) in 24‐well flat bottom plates. Heat‐killed bacteria (MOI = 1; 1 × 10^6^ bacteria/mL) were added to the cell culture medium. PHA‐L 500X (5 µg/mL; Invitrogen) and T Cell TransAct human (1:100; Miltenyi Biotec) were further used as stimulants. The cells were incubated at 37°C and 5% CO_2_ for 24 to 72 h as indicated in the respective figures.

### 4.5 PBMC Culture Stimulation with Bacterial Cell Wall Components and SCFA

PBMCs were cultured as described above. The following bacterial components were used: lipopolysaccharides (1 µg/mL, Invitrogen by Thermo Fisher Scientific), lipoteichoic acid (InvivoGen; 1 µg/mL) and peptidoglycan (InvivoGen; 10 µg/mL). Additionally, sodium‐butyrate, sodium‐acetate, and sodium‐propionate (all from Sigma‐Aldrich) were used with different concentrations as indicated in the respective figures. For time‐kinetic experiments, cells were cultured for 24, 48 and 72 h and at each respective timepoint, supernatants were collected and frozen at ‐80°C for later analysis via enzyme‐linked immunosorbent assay (ELISA). For dose‐kinetic experiments, isolated PBMCs were stimulated with LPS, LTA, and PGN in combination with SCFAs in varying concentrations (between 0.0002 and 20 mM). Stimulated PBMCs were cultured for 24, 48 and 72 h and supernatants were collected at each timepoint and frozen at −80°C for later analysis via ELISA.

### 4.6 Cell Fraction Experiments

Monocytes were isolated from human PBMCs with magnetic beads using CD14 MicroBeads from Miltenyi Biotec according to the manufacturer's protocol. Monocyte purity was above 95% as assessed by flow cytometry (FITC Mouse Anti‐Human CD14). The magnetic separation resulted in two fractions: pure monocytes and monocyte‐depleted PBMCs (as depicted in Figure 3). Both fractions were resuspended in RPMI Medium 1640 (Thermo Fisher) with 10 % heat‐inactivated FBS (Thermo Fisher). The concentration of monocytes was 2 × 10^5^ cells/mL and the concentration of monocyte‐depleted PBMCs was 1 × 10^6^ cells/mL. The cells were seeded in 24‐well flat‐bottom plates. Each fraction (PBMCs, monocytes, monocyte‐depleted PBMCs) was incubated with LPS (Invitrogen by Thermo Fisher Scientific; 1 µg/mL), LTA (InvivoGen; 1 µg/mL), LPS (Invitrogen by Thermo Fisher Scientific; 1 µg/mL) and sodium‐butyrate (Sigma‐Aldrich; 2 mM) + LTA (InvivoGen; 1 µg/mL). The supernatants were collected after 24, 48 and 72 h of stimulation and frozen at ‐80°C for later analysis by ELISA.

### 4.7 Preparation of Platelet‐Poor‐Plasma and Platelet‐Rich‐Plasma

Venous blood was drawn into sodium‐citrate tubes and gently mixed by inversion. The collection tubes were centrifuged at 300*g* for 10 min at room temperature. The plasma was transferred to an Eppendorf tube and centrifuged a second time at 700*g* for 17 min at room temperature. The PPP was obtained by taking the upper third of the plasma. The second third was discarded. The lowest third including the pellet was used as PRP. 100 µL of PPP and PRP were directly used for the cell culture experiments. The remaining plasma samples were frozen at −80°C for later analyses of the BDNF content via ELISA.

### 4.8 PRP and PPP Cell Culture Experiments

PBMCs, PRP, and PPP were prepared as described above from the same donor. PRP (100 µL) and PPP (100 µL) were added to PBMCs alone or in combination with LPS (Invitrogen by Thermo Fisher Scientific; 1 µg/mL). The supernatant was harvested after 72 h and frozen at −80°C for later analysis by ELISA.

### 4.9 PBMC‐Platelet Co‐Culture Experiment

After venous blood had been drawn into heparinized blood collection tubes, the concentration of platelets was measured using a Sysmex blood cell counter. The whole blood was centrifuged at 800*g* for 5 min at room temperature and 1 mL of plasma was collected and stored at room temperature. The PBMCs were isolated according to the prior described procedure. Before resuspending the cells in media, the cells were washed two more times in DBPS^−/−^ (Gibco) to reduce the number of platelets in the final cell suspension, which was then distributed on a 24‐well flat‐bottom plate. The platelets in the plasma were added to the cells in increasing concentrations (10^4^/mL–10^7^/mL)). Cells with added platelets were also stimulated with LPS (Invitrogen by Thermo Fisher Scientific; 1 µg/mL) and LTA (InvivoGen; 1 µg/mL) with adenosine‐triphosphate (InvivoGen; 0,5 mM). The cells were incubated, and the supernatant was collected and frozen after 24, 48, and 72 h for later analysis via ELISA.

### 4.10 Maintenance and Culture of Human Pluripotent Stem Cells

The human ESC line WA09, alias H9 (WiCell), was obtained from the laboratory of Markus Hengstschläger (Medical University of Vienna, Austria) where it was maintained under feeder‐free conditions on growth factor‐reduced Matrigel (Corning) in mTeSR1 medium (STEMCELL Technologies). Cells at passage 28 (p28) were then transitioned to mTeSR Plus medium (STEMCELL Technologies) and LN521 (BioLamina). During the entire process of propagation, cells were passed as small clumps with Versene (Lonza or ThermoFisher) and maintained at 37°C and 5% CO_2_ with daily medium changes. Experimentation was performed with cells at p30.

### 4.11 Microglia Differentiation

ESCs were differentiated into microglia as described previously by Haenseler et al. [36] In brief, colonies were dissociated with Accutase (Sigma‐Aldrich) for 10 min and 3 × 10^6^ cells in mTeSR Plus medium containing 10 µM Y‐27632 were seeded into Anti‐Adherence Rinsing Solution pre‐treated wells of an AggreWell plate (all STEMCELL Technologies) to generate embryoid bodies (EB). In the following 3 days, 75% medium exchanges were carried out with mTeSR Plus containing 50 ng/mL BMP4, 50 ng/mL VEGF_165_ and 20 ng/mL SCF (all Proteintech) to induce mesoderm differentiation. On day 4, EBs were harvested and transferred into tissue‐culture flasks (1 EB/cm^2^) in X‐VIVO 15 (Lonza) containing 1X GlutaMAX (ThermoFisher), 55 µM β‐mercaptoethanol (Sigma‐Aldrich), 100 ng/mL M‐CSF and 25 ng/mL IL‐3 (both Proteintech) for myeloid differentiation induction. Fresh medium was added every 7 days until primitive macrophage progenitors (PMPs) emerged in the supernatant. PMPs were then collected and used for microglia differentiation, while flasks containing differentiating EBs were replenished with fresh medium and returned to the incubator until the next round of harvest. For the differentiation of microglia, collected PMPs were strained (37 µm) and plated into tissue culture flasks (5 × 10^4^ cells/cm^2^) in Advanced DMEM/F12 (ThermoFisher) supplemented with 1X N2 (ThermoFisher), 1X GlutaMAX, 55 µM β‐mercaptoethanol, 100 ng/mL IL‐34, and 10 ng/mL GM‐CSF (both Proteintech). Medium was exchanged every 3 days for a total of 14 days. On day 15, MGLs were dissociated with Accutase for 15 min and were resuspended in a fresh medium for stimulation experiments.

### 4.12 Stimulation of Microglia‐Like Cells

MGLs were transferred into 24‐well plates at 1 × 10^5^ cells/cm^2^. The next day, cells were stimulated for 24 h by adding TLR‐4‐specific agonist LPS (Invivogen) at 100 ng/mL into the medium. At the end of each experiment, conditioned media were collected, cleared from cells by centrifugation at 1000*g* and 4°C for 10 min and assessed for cytokine secretions.

### 4.13 Immunocytochemistry

After 14 days of differentiation, MGLs were dissociated as described above and were seeded into 5 µg/cm^2^ PDL‐coated (Merck) removable three‐well chamber slides (Ibidi) at 1 × 10^4^ cells/cm^2^. One day later, cells were washed three times with HBBS and were fixed with 4% (w/v) PFA (ThermoFisher) for 10 min followed by three washes. Then, cells were permeabilized for 10 min with Permeabilization Buffer consisting of DPBS^−/−^ supplemented with 0.1% (v/v) Triton X‐100 (Sigma‐Aldrich), washed again three times and blocked for 1 h with Blocking Buffer (DPBS^−/−^ + 5% (v/v) normal goat serum + 0.1% (v/v) Tween20). After three washes, rabbit α‐IBA1 (5 µg/mL, Proteintech) and rat α‐TREM2 (1 µg/mL, Bio‐Techne) antibodies diluted in antibody dilution buffer made up of DPBS^−/−^, 1% (w/v) BSA (Sigma‐Aldrich) and 0.1% (v/v) Tween20 were added for 3 h. The solutions were decanted, cells were washed three times and goat anti‐rat Alexa Fluor 488 plus goat anti‐rabbit Alexa Fluor 594 conjugated secondary antibodies (both ThermoFisher) at 1:1000 dilutions were incubated for 1 h in the dark. After secondary staining, cells were washed again three times and were counterstained with DAPI (Sigma‐Aldrich) at 1 µg/mL for 5 min. Finally, cells were rinsed three times with ultrapure water and were let to dry, before mounting with a coverslip and ProLong Glass Antifade Mountant (ThermoFisher). All incubations were carried out at room temperature and washed with DPBS^−/−^, if not otherwise stated. Immunofluorescent stainings were visualized and images were captured on an LSM 980 confocal microscope (ZEISS). To avoid nonspecific bleed‐through, fluorochromes were all excited and detected independently. Image analyses were performed with Fiji/ImageJ2.

### 4.14 Enzyme‐Linked Immunosorbent Assay

The preparation work for measuring the proteins in our samples was done according to the instructions of the different ELISA kits. The DuoSet Human/Mouse BDNF and the Human Pro‐BDNF Kit (both R&D Systems) were used for BDNF/Pro‐BDNF detection. Further, ELISA kits used were for the detection of human IFN‐γ, human IL‐8, human IL‐10, and human TNF‐α (all from Invitrogen). The measurement of optical density at 450 and 570 nm was measured with the plate reader Epoch (BioTek).

### 4.15 Quantitative Polymerase Chain Reaction

PBMCs were isolated and stimulated with LPS (Invitrogen by Thermo Fisher Scientific; 1 µg/mL), LTA (InvivoGen; 1 µg/mL) and LPS (Invitrogen by Thermo Fisher Scientific; 1 µg/mL) + sodium‐butyrate (Sigma‐Aldrich); 2 mM). The cells were collected after 2, 6, 24 and 48 h and centrifuged at 300*g* for 10 min at room temperature. The cell pellet was resuspended in 200 µL of RNA stabilizing solution (RNAlater solution; Invitrogen) and frozen at −80°C. The RNA isolation was done according to the instructions of the QIAamp RNA Blood Mini Kit and the RNA concentration was measured with the Qubit4 Fluorometer (Invitrogen). For the reverse transcription, one reaction tube was prepared for each sample containing a premixed master mix (Thermo Fisher), the RNA sample and water. The samples were loaded onto the Eppendorf EP Gradient S Thermocycler (10 min at 25°C, 120 min at 37°C, 5 min at 85°C and at hold at 4°C). For the qPCR, the cDNA was mixed with the previously prepared master mix containing primers for BDNF (Hs00538277_m1; Thermo Fisher), for ACTB VIC (Hs01060665_g1; Thermo Fisher), and for 18‐S Vic PL (Hs99999901_s1; Thermo Fisher). The samples were applied in triplicates and the amplification took place in 40 cycles on the QuantStudio 5 (Thermo Fisher).

### 4.16 Apoptosis Assay

PBMCs were isolated and cultured as described above. The cells were stimulated with sodium‐butyrate (Sigma‐Aldrich; 0.0002, 0.2, and 20 mM) and with LPS (Invitrogen by Thermo Fisher Scientific; 1 µg/mL). After 72 h, the cells were harvested and rinsed with 1 mL of ice‐cold 1X DPBS^−/−^ (Gibco). The cells were centrifuged for 5 min at 300*g* and 4°C. The supernatant was discarded, and the cell pellet was resuspended in 500 µL cold 1X annexin binding buffer. Annexin V V450 (BD Biosciences) and 7‐AAD (Invitrogen by Thermo Fisher Scientific) were added to each sample. The cells were incubated for 10 min at room temperature in the dark. Afterwards, the cells were analyzed via flow cytometry (CytoFLEX S, Beckman Coulter).

### 4.17 Intracellular BDNF Detection

PMBCs were isolated and stimulated according to the stimulation schemes. At given timepoints, the cells were harvested and rinsed with 1 mL ice‐cold 1X DPBS^−/−^ (Gibco). The samples were centrifuged at 400*g* for 5 min at 4°C. The cell pellet was resuspended in 100 µL of cold staining buffer (1 X DPBS^−/−^ (Gibco) with 1% FBS (Gibco)) and 5 µL of FcR blocking buffer (Miltenyi Biotec) was added and incubated for 5 min. The following antibodies were used for the surface master mix: staining panel 1: Anti‐human CD3—Alexa Fluor 700 (Invitrogen), Anti‐human CD4—eFluor 506 (eBioscience), Anti‐human CD8a—APC‐eFluor 780 (Invitrogen), Anti‐Human CD14‐BV421 (BDHorizon), Anti‐human CD56—FITC (BioLegend); staining panel 2: Anti‐human CD3—Alexa Fluor 700 (Invitrogen), Anti‐human CD4—eFluor 506 (eBioscience), Anti‐human CD8a—APC‐eFluor 780 (Invitrogen), Anti‐Human HLA‐DR—500 (BD Horizon). The mix was added to each sample and those were incubated for 20 min in the dark. Next, 3 mL of staining buffer (1 X DPBS^−/−^ (Gibco) with 1% FBS (Gibco)) were added, and the tubes were centrifuged at 400*g* for 5 min. The cell pellet was resuspended in 50 µL of 100% FBS (Thermo Fisher). The fixation, permeabilization, and staining were done according to the instructions of the PerFix‐nc kit (Beckman Coulter). The intracellular antibody Anti‐human BDNF—PE‐conjugated (R&D Systems) was added after the permeabilizing reagent and incubated for 60 min. After the fixation, the cells were analysed directly via flow cytometry. Monocyte and NK cell populations showed no sufficient positive signal after stimulation, therefore only CD4^+^ and CD8^+^ were further analyzed using fluorescence markers. We further defined CD3^−^ lymphoid cell populations as well as CD3^−^ Monocyte Scatter populations using scatter properties as highlighted in Figure S4.

### 4.18 Imaging Flow Cytometry

After the isolation of PBMCs and monocytes, their concentration was set to 2 × 10^6^ cells/200 µL for PBMCs and 3.2 × 10^5^ cells/100 µL for monocytes in 1x DPBS^−/−^ (Gibco). The antibody master mix was prepared by combining Anti‐Human CD14 ‐ BV421 (BD Horizon), CD41 ‐ PE‐Cy7 (BIOCYTEX) and CD62p‐FITC (eBioscience)). The master mix was added to the cell suspension and the cells were stained for 20 minutes at room temperature in the dark. The samples were washed with 3 mL of 1X DPBS^−/−^ (Gibco) and the cell pellet was finally resuspended in 150 µL fixative solution IO3 (Beckman Coulter). The cells were analysed on the AMNIS Image Stream X Mark II Imaging Flow Cytometer (Luminex).

### 4.19 Statistical Analysis

R version 4.2.2 was used for data analysis and visualization using ggplot2 (version 3.4.2) and rstatix (version 0.2.7). Flow cytometry data were analysed with the software FlowJo (10.8.1) and imaging flow cytometry data were analysed with the software IDEAS (version 6.2).

## Author Contributions

Conceptualization: Fabien Sarcletti and Lukas Wisgrill. Data curation: Fabien Sarcletti, Marco Dijmarescu, Michael Eigenschink, David Seki, and Lukas Wisgrill. Formal analysis: Fabien Sarcletti, Marco Dijmarescu, Michael Eigenschink, and Lukas Wisgrill. Funding acquisition: Angelika Berger, David Berry, and Lukas Wisgrill. Investigation: Fabien Sarcletti, Marco Dijmarescu, Nadja Wukowits, Barbara Oehler, Anastasia Tandecki, Sarah Pell, Anastasia Tandecki, and David Seki. Methodology: Fabien Sarcletti, Andreas Spittler, David Berry, and Lukas Wisgrill. Project administration: Angelika Berger, David Berry, and Lukas Wisgrill. Resources: Angelika Berger, David Berry, and Lukas Wisgrill. Software: Michael Eigenschink and Lukas Wisgrill; Supervision: Lukas Wisgrill. Visualization: Fabien Sarcletti, Michael Eigenschink, Marco Dijmarescu, and Lukas Wisgrill. Writing–original draft preparation and writing–review & editing: Fabien Sarcletti and Lukas Wisgrill.

## Conflicts of Interest

The authors declare no conflicts of interest.

## Supporting information



Supporting Information

## Data Availability

The data are available from the corresponding author upon reasonable request.
