# Supplementary material for: Monocyte‐Platelet Aggregates Are Major Source of BDNF after Bacterial Stimulation of Human Peripheral Blood Immune Cells
Source: Eur J Immunol. 2025 Apr 1;55(4):e202451538. doi: 10.1002/eji.202451538 (PMC11962235; doi:10.1002/eji.202451538)
Supplement: Supplementary file 1 — Supporting Information [file EJI-55-e202451538-s001.pdf]

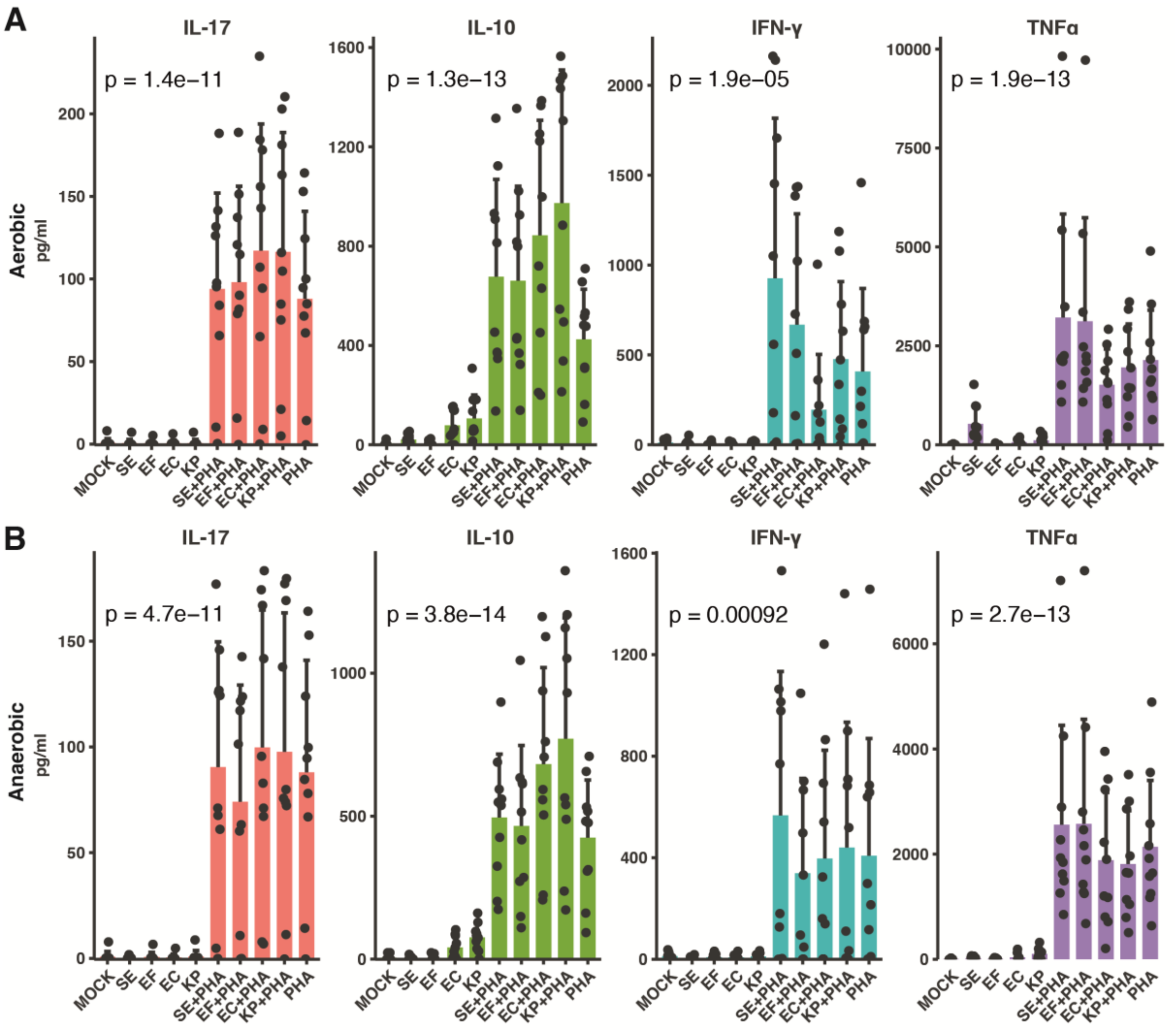

Supplemental Figure 1. Cytokine secretion after bacterial stimulation of human PBMCs. The secretion of TNFα, IFN-γ, IL-17, IL-10, and BDNF after concomitant stimulation of PBMCs ($1x10^6$ cells, n=10 per stimulation, 10 independent experiment) with phytohemagglutinin (PHA; 5 µg/ml) and heat-killed *S. epidermidis* (SE), *E. faecalis* (EF), *E. coli* (EC), and *K. pneumoniae* (KP, each $1x10^6$) grown under aerobic (A) and anaerobic (B) conditions. Cells were stimulated for 24 hours and cytokine secretion measured via ELISA. Bar graphs represent the mean concentration with SD (pg/ml). The Kruskal-Wallis-Test was used for statistical analysis.

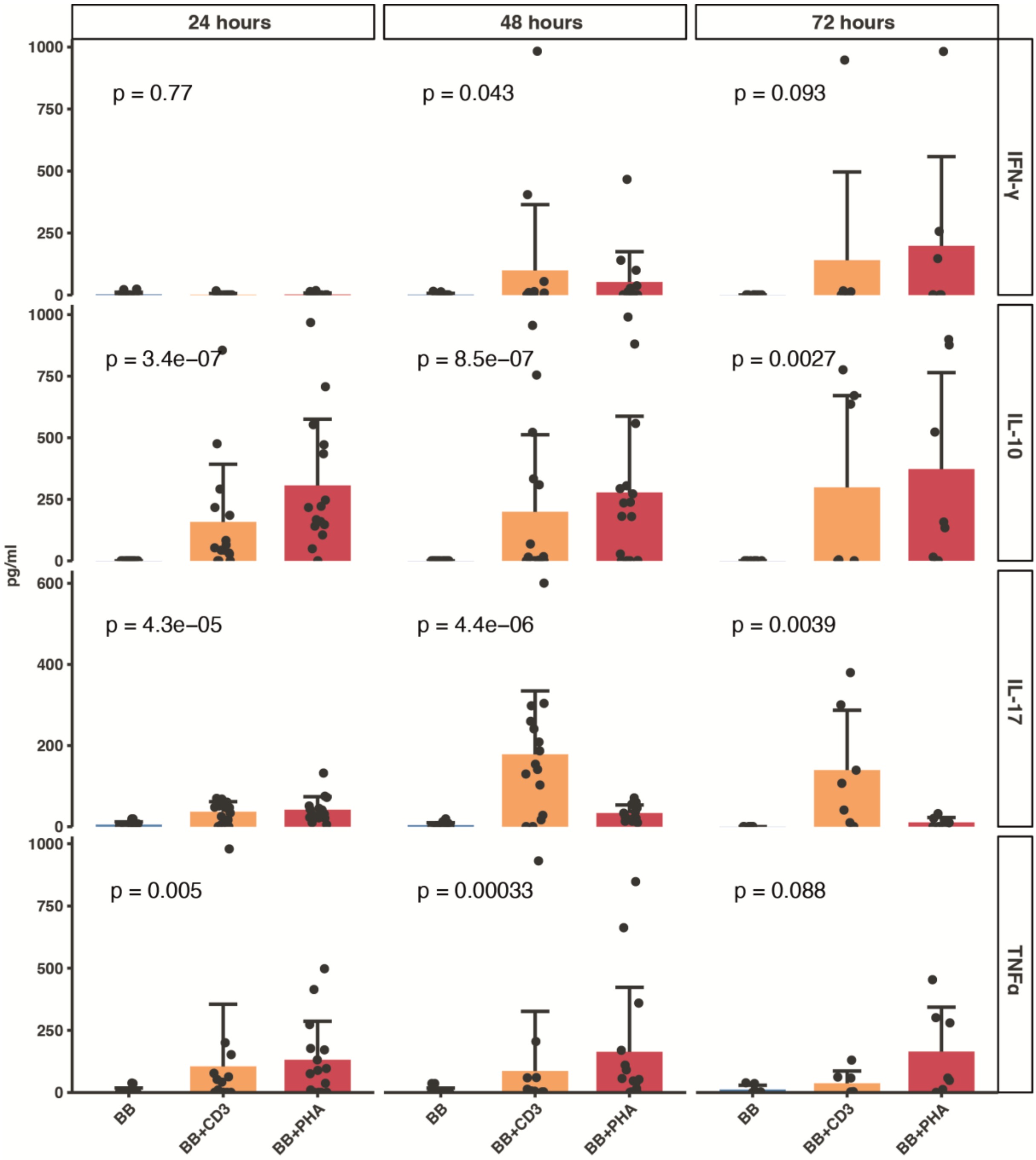

Supplemental Figure 2. Cytokine secretion after bacterial stimulation with *Bifidobacterium breve (BB)* of human PBMCs. PBMCs (n=6-15, 8 independent experiments) were stimulated with *B. breve* (BB; $1x10^6$) as well as PHA (5 µg/ml) or CD3/CD28 (CD3; 10 µg/ml) and cytokine as well as BDNF secretion was analyzed after 24, 48, and 72 hours via ELISA. Bar graphs represent the mean concentration with SD (pg/ml). The Kruskal-Wallis-Test was used for statistical analysis.

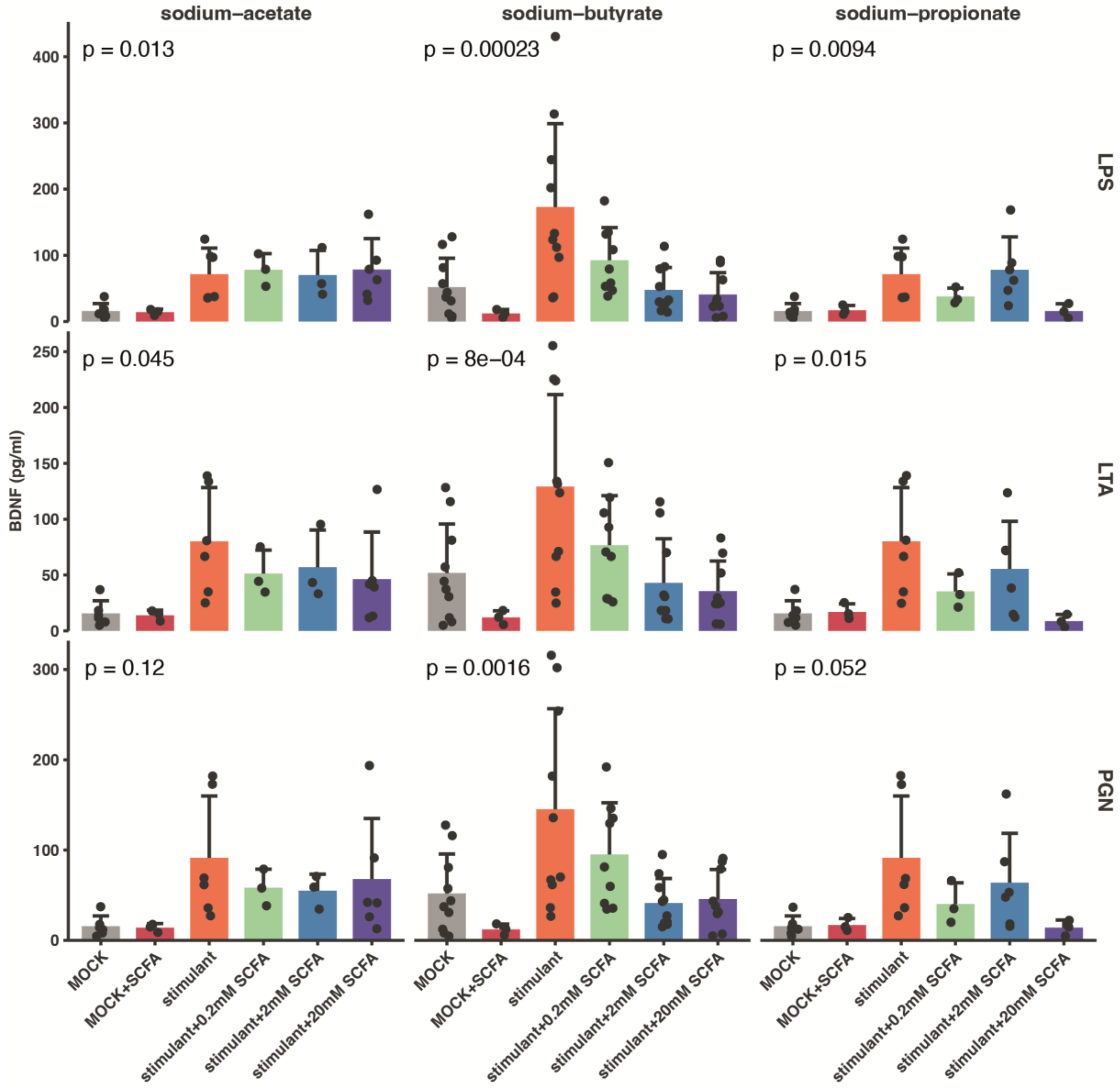

Supplemental Figure 3. Cytokine secretion after bacterial antigen stimulation with short chain fatty acids of human PBMCs. BDNF concentration after co-stimulation of PBMCs (n=3-10, 4 independent experiments) with increasing doses of sodium-acetate (left panel), sodium-butyrate (middle panel), and sodium-propionate (right panel), as well as LPS (1µg/mL; upper panel), LTA (1µg/mL, middle panel), or PGN (10µg/mL, lower panel). Cells were stimulated for 72 hours and BDNF was measured via ELISA. Bar graphs represent the mean concentration with SD (pg/ml). ANOVA was used for statistical analysis.

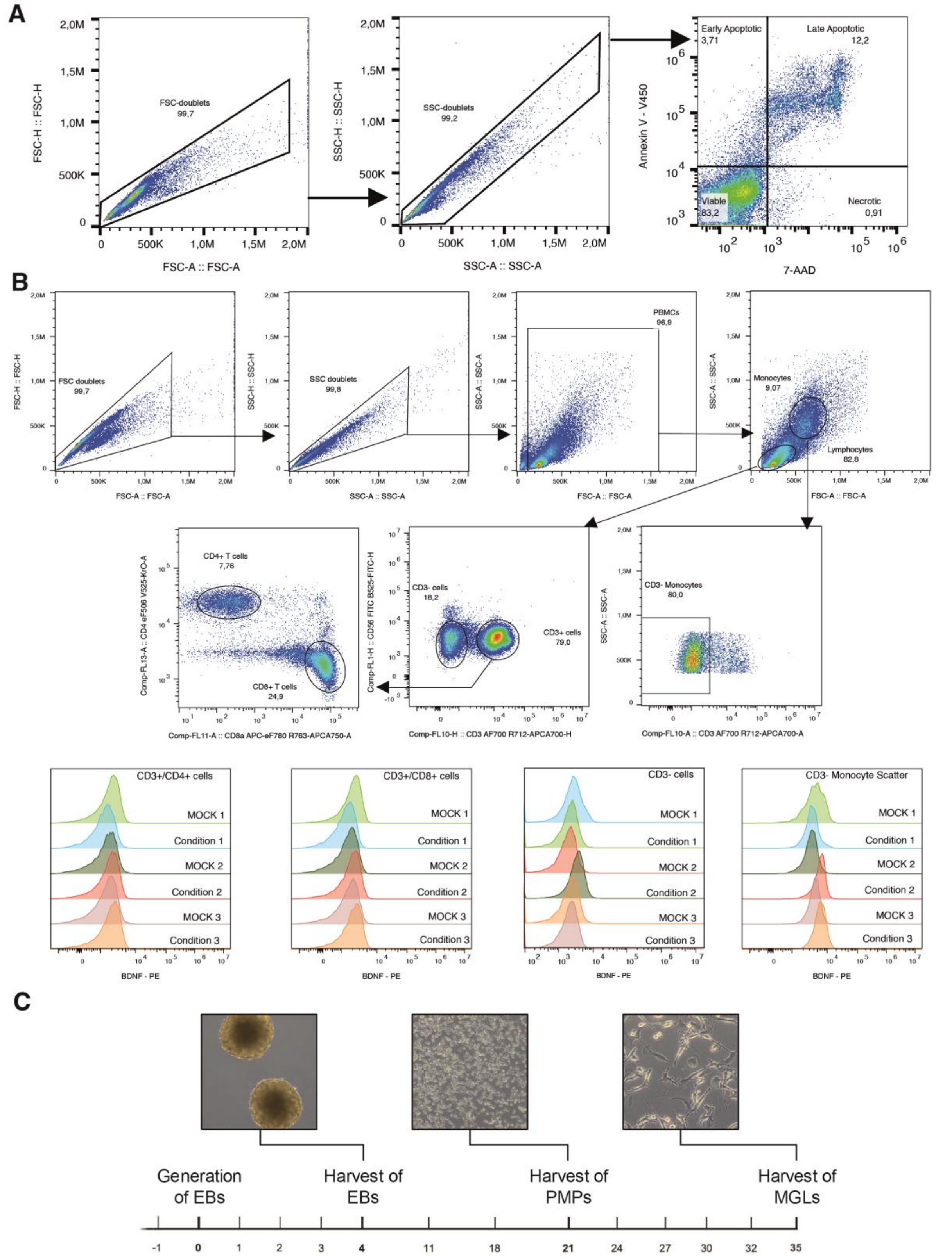

Supplemental Figure 4. Gating strategy and microglia generation overview. Used gating strategy for the apoptosis assay (A) as well as the intracellular BDNF staining panel 1 (B). Figure were generated using FlowJo. (C) Human microglia were generated *in vitro* from the human embryonic stem cell line WA09. The differentiation process

involved sequential stages, including embryoid body (EB) formation, primitive macrophage progenitor (PMP) development, and maturation into microglia-like cells (MGLs) over 35 days.
